# Assessment of baseline bone turnover marker levels and response to risedronate treatment: Data from a Japanese phase III trial

**DOI:** 10.1016/j.bonr.2020.100275

**Published:** 2020-04-25

**Authors:** Taro Mawatari, Satoshi Ikemura, Gen Matsui, Takahiro Iguchi, Hiroaki Mitsuyasu, Shinya Kawahara, Masayuki Maehara, Ryoichi Muraoka, Yukihide Iwamoto, Yasuharu Nakashima

**Affiliations:** a Department of Orthopedic Surgery, Hamanomachi Hospital, Fukuoka, Japan; b Department of Orthopedic Surgery, Graduate School of Medical Sciences, Kyushu University, Fukuoka, Japan; c Alliance Management Department, EA Pharma Co., Ltd., Tokyo, Japan; d Data Science Group, Clinical Development Department, EA Pharma Co., Ltd., Tokyo, Japan; e Department of Orthopedic Surgery, Kyushu Rosai Hospital, Fukuoka, Japan

**Keywords:** BTMs, bone turnover markers, BAP, bone isoforms of alkaline phosphatase, P1NP, N-propeptide of type I collagen, CTX, C-telopeptide of type I collagen, DPD, deoxypyridinoline, TRACP-5b, tartrate-resistant acid phosphatase-5b, SD, standard deviation, ULN, upper limit of the normal range, BMD, bone mineral density, LS-BMD, lumbar spine bone mineral density, C, central, A, anterior, P, posterior, Bone turnover markers, Bone isoforms of alkaline phosphatase, C-telopeptide of type I collagen, Tartrate-resistant acid phosphatase-5b, Risedronate

## Abstract

**Background:**

Risedronate increases bone mineral density (BMD) and reduces fracture risk, but treatment response may depend on the baseline state of bone turnover. Data regarding the selection of therapeutic drugs or the prediction of therapeutic effects with baseline levels of bone turnover markers (BTMs) as a reference are insufficient. We hypothesized that when the baseline levels of BTMs are higher, baseline BMD might be lower, changes in BMD at 12 months after risedronate treatment might be higher, and the reduction of fracture incidence might be greater. This study aimed to analyze the data of a phase III clinical trial of risedronate from Japan to investigate the relationships between baseline BTM levels and (1) baseline BMD, (2) changes in BMD at 12 months after the start of treatment, and (3) the incidence of new vertebral fractures.

**Methods:**

This post-hoc analysis included 788 postmenopausal women with osteoporosis whose baseline BTM levels as well as baseline and endpoint BMDs were measured. Relationships between baseline BTM levels and BMD at baseline and 12 months after risedronate treatment and new vertebral fractures were examined. One-way analysis of variance, two-tailed Student's *t*-test, and Fisher's exact test were used to analyze the data.

**Results:**

Baseline BMD showed a significant upward trend when baseline BTM levels were lower in the analysis by tertiles. New vertebral fractures tended to occur in patients with prevalent vertebral fractures, but the relationship between new fractures and BTM levels was not statistically significant. Regardless of BTM types, BMD percentage increments (%) and increments (g/cm^2^) with the 12-month treatment were high when pretreatment BTM levels were high (P < 0.0001), and a >5.0% increase in BMD was observed even if baseline BTM levels were within the normal range. A new vertebral fracture occurred in only six patients (0.77%), and there was not enough statistical power to clarify the relationship between baseline BTM levels and fracture risk reduction.

**Conclusions:**

When pretreatment BTM levels increased, baseline BMD tended to be lower and the increase in BMD with 12-month risedronate treatment was higher. However, BMD could still be increased even if the baseline BTM levels are within the normal range. Combined with available evidence, baseline BTMs may not have an important role in deciding the optimal therapy. To elucidate the relationship between baseline BTM levels and long-term fracture risk, it will be necessary to conduct more large-scale studies with a longer follow-up period in severe osteoporotic patients with a high fracture risk.

**Mini abstract:**

We evaluated the significance of baseline bone turnover markers in the response to risedronate treatment. The increase in the bone mineral density (BMD) with the 12-month treatment may be higher when the state of bone turnover at baseline is higher, and BMD could still be increased even if the baseline bone turnover is within the normal range.

## Highlights

• Baseline bone turnover markers in response to risedronate treatment are unknown.
• Higher BMD may be achieved when the state of bone turnover at baseline is higher.
• Further increase in BMD is possible even if the baseline bone turnover is normal.

## 1 Introduction

Bone turnover markers (BTMs) in the serum, plasma, and urine are considered useful for individual treatment monitoring and identification of poor adherence to both antiresorptive and osteoanabolic agents in clinical practice (Eastell et al., 2018; Tsujimoto et al., 2011). Bone formation markers include bone-specific alkaline phosphatase (BAP), N-propeptide of type I collagen (P1NP), and osteocalcin, whereas bone resorption markers include serum or urinary N-telopeptide of type I collagen, serum and urinary C-telopeptide of type I collagen (CTX), urinary deoxypyridinoline (DPD), and serum enzyme tartrate-resistant acid phosphatase-5b (TRACP-5b) (Eastell et al., 2018).

The values of bone resorption markers >1.0 standard deviation (SD) above the mean in healthy premenopausal women have been reported to indicate a high rate of bone loss (Delmas, 2000; Nishizawa et al., 2005). However, in osteoporotic patients who have low bone mass, BTMs have not been shown to be predictive of future bone mass changes (Delmas, 2000). In a prospective epidemiologic study, high BTM levels were reported to be related to an increase in fragility fracture risk (i.e., vertebral and femoral neck fractures). In cases where bone resorption markers show values above the upper limit of the normal range (ULN > 1.96 SD above the mean in healthy premenopausal women), a high future fracture risk has been reported (Nishizawa et al., 2005); however, sufficient consensus on this issue has not been achieved to date.

Regarding the baseline condition and treatment effect of bisphosphonates, Mawatari et al. (2017) explored the influence of baseline age, bone mineral density (BMD), and serum 25(OH)D concentration on the response to risedronate by analyzing the phase III clinical trials of risedronate in Japan (CCT-003, 101 and 301) (Mawatari et al., 2017). As a result, the risedronate-induced increase in BMD was consistent across all age tertiles and was lower in patients with vitamin D deficiency.

Several reports have been published on the relationship between changes in BTMs after risedronate therapy and future increases in BMD and the incidence of fractures (Eastell et al., 2003; Mawatari et al., 2017; Raisz et al., 2000). However, only few reports exist on the investigation of the relationship between baseline BTM levels and effects of risedronate therapy. Seibel et al. (2004) reported on the relationships between baseline levels of urinary deoxypyridinoline and baseline BMD, future increases in lumbar spine BMD with risedronate treatment, and incidence of vertebral fractures; however, they only divided the levels of BTMs according to their baseline median values and did not study other markers or levels higher or lower than the ULN. Okazaki et al. (2019) have shown that low BTM levels, low serum 25(OH)D concentration, and high BMD at baseline were independent predictors of an inadequate response to risedronate in a qualitative manner by analyzing phase III clinical trials of risedronate in Japan (CCT-101 and 301).

Intuitively, antiresorptives, such as bisphosphonates, appear to be recommended for patients with increased bone resorption (Bauer et al., 2006; Seibel, 2006; Vasikaran et al., 2011). However, data for the selection of therapeutic drugs or the prediction of therapeutic effects with baseline levels of BTMs as a reference are insufficient (Bergmann et al., 2009; Eastell et al., 2018; Reginster et al., 2008; Vasikaran and Chubb, 2016). In a fracture intervention trial of alendronate, the treatment was more effective at reducing non-vertebral fracture women with a higher P1NP level, but this was not true for other BTMs or other fracture types (Bauer et al., 2006). A low P1NP level is associated with lower rates of bone loss and lower BMD response to zoledronic acid (Eastell et al., 2015). Therefore, it would be interesting to know the extent of the difference in the effects of other antiresorptives, such as risedronate, between patients with moderate increases in bone resorption and those with high increases in bone resorption.

We hypothesized that when the baseline levels of BTMs are higher, baseline BMD might be lower, changes in BMD at 12 months after risedronate treatment might be higher, and the reduction of fracture incidence might be greater. Moreover, the objectives of this study were to analyze the data of a phase III clinical trial of risedronate use from Japan to investigate the relationships between baseline levels of BTMs and (1) baseline BMD, (2) changes in BMD at 12 months after the start of treatment, and (3) incidence of new vertebral fractures.

## 2 Materials and methods

### 2.1 Study design

This study analyzed the data from a randomized, double-blind clinical phase III trial for risedronate, namely, CCT-301 (Hagino et al., 2014). This trial was performed as a multicenter study in Japan between February 2010 and August 2011. The primary efficacy endpoint was the percent change in the mean lumbar spine BMD (LS-BMD).

Trial CCT-301 involved 12 months of treatment wherein eligible patients were randomly assigned to receive either a monthly (75 mg) or daily (2.5 mg) oral dose of risedronate. Blinding to the study drug was maintained by a double-dummy technique using active drugs and corresponding placebo tablets.

All patients were treated in an ambulatory setting and were supplemented with calcium lactate (1.54 g/day, corresponding to 200 mg of calcium per day) throughout the study period. However, vitamin D was not administered as a supplement to ensure consistency with previous clinical trials. In addition, concomitant use of any drugs known to affect bone metabolism was prohibited.

The study protocol was approved by the institutional review board of each institution before study initiation, and all patients provided written informed consent before registration. The trial was conducted according to the Good Clinical Practice guidelines and principles of the Declaration of Helsinki. Details of the study design, patients involved, and protocols of this trial have been described elsewhere (Hagino et al., 2014).

### 2.2 Patient selection

**Fig. 1:**
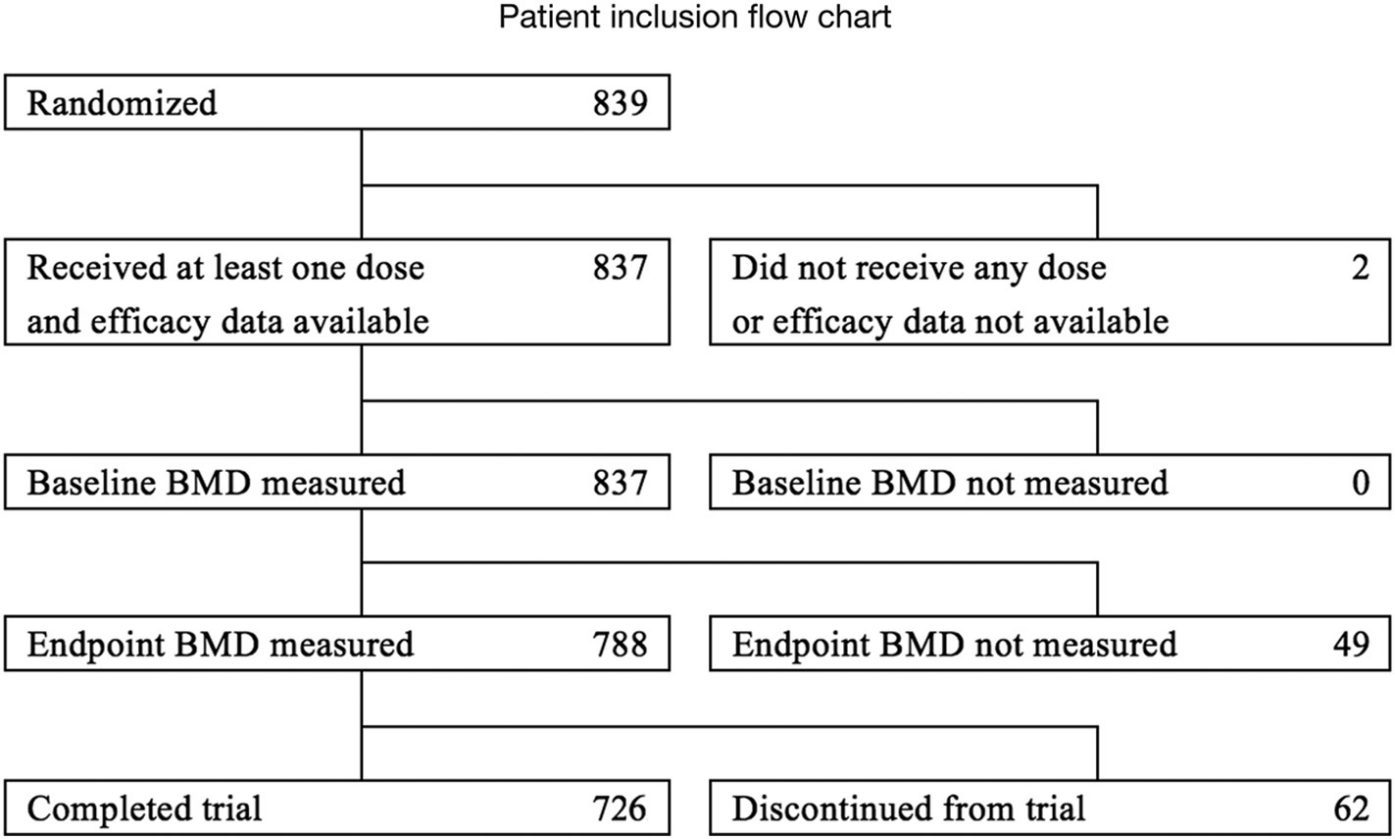
Patient inclusion flowchart. Data were collected from a randomized, double-blind, clinical phase III trial for risedronate. BMD, bone mineral density.

The present study consists of a post-hoc analysis of data from 788 postmenopausal women with osteoporosis who received risedronate treatment (Fig. 1).

Ambulatory patients of either sex, aged ≥50 years, with involutional osteoporosis were eligible if they met the diagnostic criteria for primary osteoporosis established by the Japanese Society for Bone and Mineral Research (Orimo et al., 1998, Orimo et al., 2001). Primary osteoporosis was defined as the presence of a fragility fracture and BMD <80% of the “young adult mean” (aged 20–44 years) or BMD <70% of the “young adult mean” in the absence of a detectable fragility fracture. The exclusion criteria were as follows: secondary osteoporosis or other diseases known to reduce bone mass; radiographic findings that might affect the LS-BMD; recent use of drugs known to affect bone metabolism; gastrointestinal diseases; hypocalcemia; hypercalcemia; serious renal, hepatic, or cardiac diseases; malignant tumors under treatment with antitumor agents; drug hypersensitivity; and history of radiotherapy to the lumbar spine or pelvis (Fig. 1).

### 2.3 Clinical examinations

The effect of risedronate treatment was evaluated in terms of LS-BMD and the incidence of new, non-traumatic vertebral fractures (including worsening of pre-existing fractures) throughout the treatment. The LS-BMD (L_2_–L_4_) was determined at baseline and designated time points via dual-energy X-ray absorptiometry using QDR-type instruments. These LS-BMD results were assessed by a specialized central review committee that was blinded to patient information.

Thoracic and lumbar spine X-rays were taken at baseline and at the end of the trial. A specialized central review committee decided whether there was pre-existing, new, or worsening of pre-existing fractures. An incident vertebral fracture was considered to have occurred if the ratio of the central to anterior vertebral height (C/A) was <0.8 or the ratio of the central to posterior vertebral body height (C/P) was <0.8; the ratio of anterior to posterior vertebral height (A/P) was <0.75, or A, C, or P decreased by at least 20% from their baseline values. A semiquantitative assessment method was used to detect worsening of an existing fracture based on whether A, C, or P had decreased by at least 20% (or 4 mm) from their baseline values, in which case the condition was labeled to have progressed by one grade or higher. There was progression by one grade or higher in these cases (Genant et al., 1993).

As biochemical markers of BTMs, serum BAP, serum TRACP-5b, and urinary CTX were assessed at baseline and after 1, 3, 6, 9, and 12 months or upon study discontinuation. Blood and urine collection were conducted at a specified time after an 8-hour fasting period for each patient. Serum and urine samples were kept frozen at ≤− 20 °C until analysis. The results of the biochemical markers of bone metabolism assays were measured at SRL (Tokyo, Japan) using standard methods. More specifically, level of serum BAP was measured by chemiluminescent enzyme immunoassay on an automatic analyzer (UniCel DxI 800, Beckman Coulter, LaBrea, CA) using the Access Ostase reagent. Serum TRACP-5b level was measured using the OSTEOLINKS® TRACP-5b kit (DS Pharma Biomedical, Osaka, Japan). Urinary CTX level was analyzed using the Urine BETA CrossLaps® ELISA kit (Nordic Bioscience Diagnostics, Herlev, Denmark) (Hagino et al., 2014).

### 2.4 Statistical analyses

To examine the significance of baseline BTMs, patients were divided into tertiles based on the level of BTMs at baseline. The baseline age, body mass index (BMI), LS-BMD (g/cm^2^), serum 25(OH)D concentrations, percentage change, and change from baseline in LS-BMD due to treatment with risedronate were evaluated in each group.

One-way analysis of variance was used to compare the baseline age, BMI, LS-BMD, and serum 25(OH)D concentrations between patient tertiles defined according to the level of BTMs. Two-tailed Student's *t*-tests were conducted to compare the new vertebral fracture incidence between the two groups with or without prevalent vertebral fracture at baseline.

Analysis of variance adjusted by possible confounders including age, BMI, baseline LS-BMD, and serum 25(OH)D was used to compare the percentage change and absolute increments in LS-BMD among patient tertiles defined according to the level of BTMs, and their least square means were calculated.

Fisher's exact tests were conducted to compare the incidence of new vertebral fractures between the patient groups defined based on BTM tertiles and the cut-off level of BTMs at baseline. Scatter plots between BTMs were drawn, and Pearson's correlation coefficients were calculated. In addition, the number of patients needed to obtain 80% statistical power for fracture risk using BTMs was calculated based on our data. All analyses were performed using SAS version 9.3 (SAS Institute Inc., Cary, NC, USA).

## 3 Results

Of the 839 randomized patients, we assessed 788 postmenopausal women with osteoporosis whose LS-BMD was measured at baseline and the endpoint (Fig. 1). The baseline characteristics of all patients included in our analysis, as well as the data in patients with or without prevalent vertebral fractures are shown in Table 1. These 788 female osteoporotic patients (age 67.8 years, BMD −3.12 SD) presented with a normal average level of serum BAP at 26.4 U/L (7.9–29.0), slightly higher average serum TRACP-5b level at 466 mU/dL (120–420), and average urinary CTX level at 319 μU/dL (40.3–301.4).

**Table 1:** Baseline characteristics of postmenopausal women with osteoporosis undergoing treatment with risedronate (n = 788).

	Overall (n = 788)			Prevalent vertebral fractures			ULN
	Mean ± SD	Median	IQR	Yes (n = 172)	No (n = 616)	P-value^a^	
				Mean ± SD	Mean ± SD		
Age (years)	67.8 ± 6.7	68.0	9.0	70.0 ± 7.0	67.2 ± 6.5	<0.0001	
Height (cm)	151.2 ± 5.6	151.0	7.0	150.0 ± 5.5	151.5 ± 5.6	0.0015	
Weight (kg)	49.5 ± 7.0	48.9	8.2	50.4 ± 6.9	49.3 ± 7.0	0.0703	
BMI (kg/m^2^)	21.7 ± 3.0	21.4	3.6	22.4 ± 3.0	21.5 ± 2.9	0.0002	
Daily/monthly oral dose of risedronate	400/388			82/90	318/298		
Lumbar spine BMD (g/cm^2^)	0.640 ± 0.063	0.650	0.087	0.644 ± 0.074	0.639 ± 0.061	0.4565	
T-score	−3.12 ± 0.54	−3.04	0.73	−3.09 ± 0.62	−3.13 ± 0.51	0.4565	
Serum 25(OH)D (ng/mL)	21.0 ± 6.8	20.0	9.0	21.2 ± 7.0	21.0 ± 6.7	0.7850	
Serum BAP (U/L)	26.4 ± 8.7	25.2	10.4	27.6 ± 11.2	26.1 ± 7.9	0.0986	29
Serum TRACP-5b level (mU/dL)	466 ± 166	444	195	483 ± 163	462 ± 166	0.1394	420
Urinary CTX level (μg/mmol·CRE)	319 ± 146	295	170	312 ± 151	321 ± 144	0.4836	301.4

Data given as mean ± standard deviation.

Upper limit of normal was defined based on reference Nishizawa et al., 2012.

BMI, body mass index; BMD, bone mineral density; BAP, bone alkaline phosphatase; TRACP-5b, tartrate-resistant acid phosphatase-5b; CTX, collagen type 1 cross-linked C-telopeptide; SD, standard deviation; IQR, interquartile range; ULN, upper limit of normal.

a Two-samples *t*-test.

Table 2 shows the baseline age, BMI, lumber spine BMD, and serum 25(OH)D concentration divided by baseline tertiles of the BTMs. Baseline LS-BMD showed a significant upward trend when the baseline level of the bone formation marker serum BAP, bone resorption marker serum TRACP-5b, or urinary CTX was lower in the analysis by tertiles (P = 0.0493, P = 0.0020, and P = 0.0274, respectively). The pretreatment level of BAP was slightly higher when the BMI was higher, and that of TRACP-5b and urinary CTX was slightly lower when the BMI was higher.

**Table 2:** Baseline characteristics by baseline tertiles of BTMs.

	Baseline serum BAP (U/L)				Baseline serum TRACP-5b (mU/dL)				Baseline urinary CTX/CRN (μg/mmol·CRE)			
	<22.2	22.2–28.8	≧28.8	P value^a^	<22.2	22.2–28.8	≧28.8	P value^a^	<22.2	22.2–28.8	≧28.8	P value^a^
Age (years)	68.1 ± 6.8	67.5 ± 6.4	67.8 ± 7.0	0.5813	67.5 ± 6.4	67.5 ± 6.8	68.5 ± 6.9	0.1411	68.6 ± 6.5	67.6 ± 6.5	67.2 ± 7.1	0.0571
BMI (kg/m^2^)	21.1 ± 2.8	21.7 ± 2.9	22.2 ± 3.1	0.0003	21.9 ± 2.8	21.9 ± 3.0	21.2 ± 3.0	0.0150	22.1 ± 3.0	21.6 ± 2.8	21.3 ± 3.0	0.0123
Lumbar spine BMD (g/cm^2^)	0.646 ± 0.065	0.642 ± 0.064	0.632 ± 0.062	0.0493	0.649 ± 0.065	0.641 ± 0.062	0.630 ± 0.063	0.0020	0.648 ± 0.061	0.638 ± 0.065	0.634 ± 0.065	0.0274
Serum 25(OH)D (ng/mL)	21.0 ± 6.8	21.2 ± 6.8	20.9 ± 6.8	0.8788	20.4 ± 6.8	20.7 ± 6.6	22.0 ± 6.9	0.0135	21.1 ± 6.9	21.1 ± 6.5	20.9 ± 7.1	0.9440

Data given as mean ± standard deviation.

BAP, bone alkaline phosphatase; TRACP-5b, tartrate-resistant acid phosphatase 5b; CTX, collagen type 1 cross-linked C-telopeptide; BMI, body mass index; BMD, bone mineral density; SD, standard deviation.

a Analysis of variance.

In addition, the baseline characteristics of the patients with BTMs <ULN were compared with those of patients with BTMs ≥ULN at baseline, and the overall trend was not different between these groups (data not shown).

New vertebral fractures tended to occur when patients had prevalent fragility fractures, although this was not statistically significant (1.74% vs. 0.49% with or without prevalent vertebral fracture at baseline, respectively. P = 0.1218). A new vertebral fracture occurred in only six patients (0.77%), and the relationships between new vertebral fractures and all BTMs were not statistically significant.

Moreover, to understand the possible effect of recent fractures in this study, the relationship between baseline BTMs and baseline LS-BMD, the relationship between new vertebral fracture and baseline BTMs, and the relationship between baseline BTMs and future increase in LS-BMD were re-examined in patients with or without prevalent vertebral fractures independently. There were some fluctuations, but the overall trend was not different between the prevalent vertebral fracture and no fracture groups (data not shown). The estimated numbers of patients needed to obtain 80% statistical power for fracture risk using BTMs were 3658, 10,658, and 3990 for serum BAP, serum TRACP-5b, and urinary CTX, respectively.

**Fig. 2:**
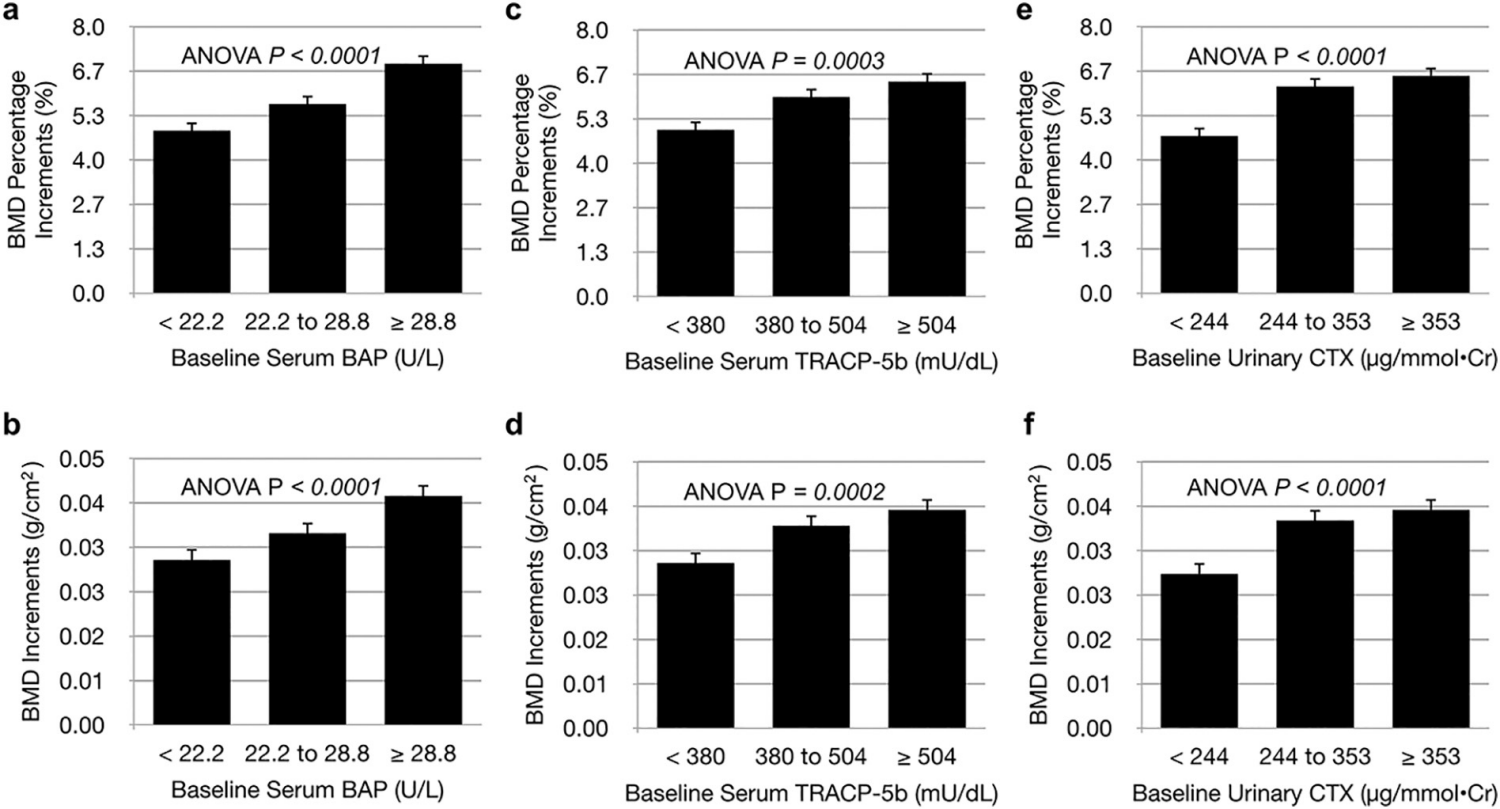
Relationships between baseline BTM levels and future increase in lumbar spine BMD adjusted f baseline age, BMD, and serum 25(OH)D concentration. Patients are divided into tertiles based on the levels of serum BAP (a, b), serum TRACP-5b (c, d), and urinary CTX (e, f) at baseline, and all data were adjusted by baseline age, BMD, and serum 25(OH)D concentrations. Error bars represent the standard error. Percentage (a, c, e) and absolute (b, d, f) increments in LS-BMD at baseline are shown. BMD, bone mineral density; BTMs, bone turnover markers; LS-BMD, lumbar spine bone mineral density; BAP, bone isoforms of alkaline phosphatase; CTX, C-telopeptide of type I collagen TRACP-5b, tartrate-resistant acid phosphatase-5b; ANOVA, analysis of variance.

Overall, the mean BMD percentage increment was 5.82% ± 4.29%, and the mean BMD increment was 0.0366 ± 0.0264 g/cm^2^. Regardless of the kind of BTMs analyzed, both BMD percentage increments (%) and increments (g/cm^2^) by risedronate were significantly higher when the pretreatment BTM levels were elevated in the analysis by tertiles (all P < 0.0001) even after adjusting for baseline age, BMI, BMD, and serum 25(OH)D concentrations (Fig. 2a–f).

Even if the BTMs were in the lowest tertile or within the normal range of premenopausal women, BMD increased by >4.6% with 12-month risedronate treatment, and differences in the BMD gain between ≥ULN and <ULN in serum BAP, serum TRACP-5b, and urinary CTX were 1.85%, 1.31%, and 1.44%, respectively.

**Fig. 3:**
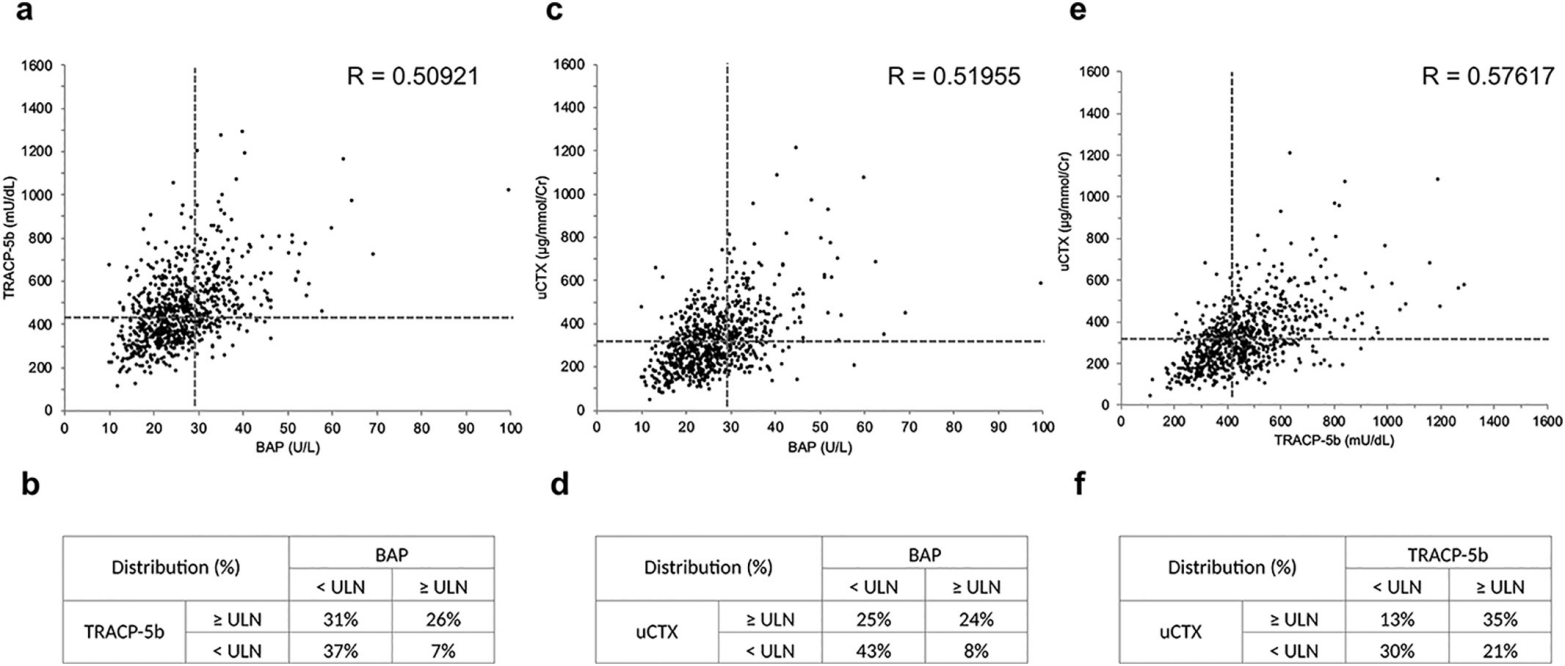
Linear regression analysis of serum BAP versus serum TRACP-5b (a), serum BAP versus urinary CTX (c), and serum TRACP-5b versus urinary CTX (e) at baseline. Distribution of patients based on four bone turnover statuses (b, d, f). Dotted lines represent the upper limit of normal of the corresponding markers. BAP, bone isoforms of alkaline phosphatase; CTX, C-telopeptide of type I collagen TRACP-5b, tartrate-resistant acid phosphatase-5b; ULN, upper limit of normal.

Fig. 3a, c, and e presents the relationships among serum BAP, TRACP-5b, and urinary CTX, respectively. Any combination of BAP and two bone resorption markers was positively correlated (R > 0.5). Moreover, the distribution of patients in this cohort was examined (Fig. 3b, d, f). When serum levels of BAP and TRACP-5b were evaluated, both bone formation and resorption were elevated in 26% of the patients, whereas, in 37% of patients, they were within the normal range.

## 4 Discussion

BTMs provide physicians with a noninvasive approach for studying bone turnover, and their levels can be measured easily with good precision. Potential clinical utilities of baseline BTMs besides monitoring therapy may include prediction of future bone loss, future fracture, future treatment response to the treatment, and selection of therapy, as well as the exclusion of abnormal bone status, such as metastatic cancer or hyperparathyroidism. In this paper, we explored the significance of the baseline BTM measurement.

### 4.1 Prediction of future bone loss

Patients with higher BTMs presented with a downward trend of BMD at baseline (Fig. 2). A long-lasting high bone turnover rate could cause bone loss. It should be noted that BTMs are vectors, and their values do not necessarily represent BMD at a specific point. However, BTMs might be useful for the prediction of future bone loss. In cohort studies consisting of only women, it was reported that the higher the BTM, the more rapid the bone loss (Eastell et al., 2018; Shieh et al., 2016).

### 4.2 Prediction of future response to the treatment

Several previous studies have reported that a bisphosphonate is most effective in increasing BMD in patients with elevated bone turnover (Bauer et al., 2006; Nishizawa et al., 2013; Seibel, 2006; Vasikaran et al., 2011). In the case of risedronate, while the use of BTMs for monitoring treatment has been discussed in several studies (Eastell et al., 2003; Raisz et al., 2000), there are few reports available on baseline BTMs and treatment effect. Seibel et al. (2004) reported that women with urinary DPD above the ULN gained LS-BMD at a faster rate than those with low DPD during the first year of treatment, although the reduction in overall fracture risk appeared to occur independent of the baseline bone turnover.

Hagino et al. (2014) reporting the results of the Japanese phase III trial of the risedronate monthly formula showed the higher increase in BMD in the highest baseline BTMs tertiles, but statistical analyses were not performed on these relationships. Okazaki et al. (2019) have shown that low serum 25(OH)D and BTM as well as high BMD at baseline were independent predictors of an inadequate responder to risedronate using a qualitative measure, but the direct relationships between baseline BTMs and the treatment effect were not analyzed. Mawatari et al. (2017) addressed that percentage, but not absolute, increments in BMD by risedronate was higher when baseline BMD was lower, which raises the question that baseline BMD might be one of the confounding factors in the relationships between baseline BTMs and increase in BMD. Therefore, we evaluated both percentage and absolute increments in BMD and compared the data adjusted by potential confounding factors, including baseline age, BMI, BMD, and serum 25(OH)D concentrations.

In our quantitative analyses, regardless of the kind of BTMs examined, both BMD percentage increments (%) and BMD increments (g/cm^2^) after a 12-month treatment with risedronate were significantly higher when the pretreatment BTM levels were higher. However, even if the BTMs were in the lowest tertile or within the normal range of premenopausal women, BMD increased by ≥4.6% with the treatment, and the effect of risedronate treatment was confirmed even in patients with a normal bone turnover.

### 4.3 Prediction of fracture risk reduction

The history of prior fracture is an important risk factor for future fracture (Klotzbuecher et al., 2000). Our results also indicated this, although the difference did not reach statistical significance. Several studies have reported that high BTM levels are associated with an increased risk of several types of fracture in men and women (Johansson et al., 2014; Vasikaran et al., 2011; Vilaca et al., 2017). However, not all studies found an association between BTMs and fracture risk (Eastell et al., 2018; Marques et al., 2016).

When baseline BTMs are higher, baseline BMD might be lower, which means that the initial fracture risk might be higher. However, an increase in BMD with 12-month risedronate treatment would be higher, and fracture risk reduction would be dependent on the duration of the evaluation period.

Conversely, a new vertebral fracture occurred in only six patients (0.77%) in our study, although the results are exploratory. According to our power analysis using this cohort, to obtain 80% statistical power for fracture risk using BTMs, we needed to collect 4000 to 10,000 patients for the trial. Recently, Crandall et al. (2018) reported a nested case-control analysis of the predictive value of BTMs for hip fracture in women participating in the Women's Health Initiative study, in which the average follow-up period was >7 years. In their study, neither the serum CTX level nor serum P1NP level was statistically significantly associated with hip fracture risk. The lack of statistical significance in both sets of analyses was probably due to the low incidence of fractures in the populations studied, minimizing the importance of these data in deciding if BTMs are useful for predicting fracture risk in response to antiresorptive treatment. Based on the available evidence, BTMs do not have an established role in the assessment of long-term fracture risk in an untreated patient, but high levels of BTMs in patients have been suggested to be partly attributed to the high imminent fracture risk observed in patients with a recent fragility fracture (Langdahl, 2018).

### 4.4 Selection of therapy

Intuitively, physicians might use antiresorptive therapies in patients with high BTM levels and anabolic therapies in patients with low BTMs. However, this approach is not supported by the results of clinical trials; BTMs did not always predict the fracture benefit with both antiresorptives and osteoanabolic agents (Bergmann et al., 2009; Eastell et al., 2018; Reginster et al., 2008; Vasikaran and Chubb, 2016). With regard to teriparatide, BMD gain by the treatment has been reported to be higher when the serum P1NP or CTX level was higher than the ULN at baseline (Yamamoto et al., 2013). This result may be because patients with high bone turnover are thought to have large numbers of remodeling units, which are called basic multicellular units (Parfitt, 1994). In other words, regardless of the antiresorptives or osteoanabolic agents, BMD gain by treatment would be higher if the baseline bone turnover was elevated, and a certain degree of BMD gain can be anticipated even when the turnover was not elevated, although the effect on the fracture risk reduction was controversial. Therefore, BTMs should not be used in individual patients when deciding on the optimal therapy (Eastell et al., 2018), and the use of osteoanabolic agents should be decided based on the severity of osteoporosis. Currently, the clinical use of BTMs is mainly for monitoring patients' response to therapy.

### 4.5 BTM selection

Three BTMs evaluated in this study were well correlated; however, urinary BTMs are affected by the time of day the samples are obtained and renal function. In clinical practice, serum-based markers, such as CTX (Vasikaran et al., 2011) or TRACP-5b, would be more useful. The International Osteoporosis Foundation/International Federation of Clinical Chemistry and Laboratory Medicine recommends one bone formation marker (serum P1NP) and one bone resorption marker (serum CTX) to be used as reference markers to compare the performance of alternatives and to expand the international experience of the application of markers to clinical medicine (Vasikaran et al., 2011).

### 4.6 Bone turnover subtypes

As shown in Fig. 3, the baseline condition of bone turnover of patients included in this clinical trial can be intuitively understandable. In our study that considered serum BAP, serum TRACP-5b, and urinary CTX, both bone formation and resorption markers were below the ULN in 37%–43% of patients and above the ULN in 24%–26% of patients (Fig. 3). Fisher et al. (2018) attempted to develop a practical model for classifying bone turnover status based on serum P1NP and serum CTX levels using their ULN and explored their clinical usefulness. They assessed orthogeriatric patients (846 women, 377 men; mean age 78.1 ± 9.50 years), and patients with low bone formation and high bone resorption (22.2%) and with elevated both markers (55.8%) were reported to be associated with in-hospital mortality. Although further research is needed to establish a subtype analysis, it is interesting to see the difference in the distribution of patients when looking at different cohorts using subtype classification. Moreover, it would be fascinating to observe the individual subtype transitions after the intervention.

The current study is limited by its post-hoc design and because no placebo groups were available for comparison in the trial. In addition, we did not exclude patients with a recent fracture, which may have increased the level of baseline BTMs, because we found that the overall trend was not different between patients with or without prevalent fractures. Moreover, there might be other potential variables to consider, but a multivariate analysis was not conducted in this study. We compared the baseline characteristics between high and low BTM groups (data not shown), and each variable was not different between the groups. Furthermore, vitamin D was not used as a supplement in this study to ensure consistency with previous trials, which might have affected the treatment response (Mawatari et al., 2017). Finally, the follow-up period of 12 months may not be sufficient to assess fracture risk prediction.

## 5 Conclusions

When the pretreatment BTM level was elevated, baseline BMD tended to be lower. Regardless of the type of BTMs, both BMD percentage increments (%) and quantitative increments (g/cm^2^) with the 12-month risedronate treatment were higher when the pretreatment levels of BTMs were elevated, whereas more than a 5.0% increase in BMD was observed even when baseline BTM levels were within the normal range. Combined with available evidence, baseline BTMs may not have an important role in deciding the optimal therapy. Future studies with a larger scale and longer observational period that include severe osteoporotic patients will be necessary to elucidate the relationship between pretreatment level of BTMs and the long-term fracture risk.

## CRediT authorship contribution statement

**Taro Mawatari:** Conceptualization, Methodology, Writing - original draft. **Satoshi Ikemura:** Visualization, Writing - review & editing. **Gen Matsui:** Validation. **Takahiro Iguchi:** Writing - review & editing. **Hiroaki Mitsuyasu:** Writing - review & editing. **Shinya Kawahara:** Validation. **Masayuki Maehara:** Data curation, Visualization, Validation, Writing - review & editing. **Ryoichi Muraoka:** Data curation, Visualization, Validation, Writing - review & editing. **Yukihide Iwamoto:** Writing - review & editing. **Yasuharu Nakashima:** Supervision.

## Declaration of competing interest

Dr. Mawatari, Dr. Ikemura, Dr. Matsui, Dr. Iguchi, Dr. Mitsuyasu, Dr. Kawahara, Dr. Iwamoto, and Dr. Nakashima have no conflict of interest to declare with respect to the work described herein. Mr. Maehara and Mr. Muraoka disclose employment relationships with EA Pharma Co., Ltd., Tokyo, Japan.
